# Supplementary material for: Risk factors for the prognosis of patients with sepsis in intensive care units
Source: PLoS One. 2022 Sep 6;17(9):e0273377. doi: 10.1371/journal.pone.0273377 (PMC9447880; doi:10.1371/journal.pone.0273377)
Supplement: S1 File — (PDF) [file pone.0273377.s003.pdf]

Clinical Research Approval Form of the Research Ethics Committee of Qinhuangdao Jungong Hospital

Project Name	The application of superior mesenteric artery ultrasound in the evaluation of gastrointestinal function in septic patients.						
Project Category	Clinical Research						
Applied Department	Intensive Care Unit	Head of Depart ment	Qiuyan Wang	Head of Project	Xiaowei Gai	Start and End Time	2020.01-2021.12
Research Purposes	Sepsis continues to be the leading cause of mortality in the intensive care unit, and still one of the top ten causes of death in the world with high morbidity and high mortality. In recent years, the gastrointestinal tract has long been hypothesized to play an integral role in the pathophysiology of sepsis. Therefore, early and efficient evaluation of gastrointestinal function may be the target for the treatment of sepsis. The main purpose of this study is to evaluate the gastrointestinal perfusion and functional status of septic patients with superior mesenteric artery ultrasound, and to take appropriate measures early to guide the direction of treatment, curb multiple organ failure, and improve long-term outcomes.						
Involving human research content	Protect the privacy of patients and respect the wishes of patients and /or their families.						
Possible adverse reaction and prevention or compensation measures	1. Strictly abide by the inclusion and exclusion criteria, and make the diagnosis based on the Sepsis 3.0 Criteria to avoid missed diagnosis and misdiagnosis;<br>2. Ultrasound examination is non-invasive and harmless, but special circumstances such as allergy to couplant should be noted. Therefore, strict screening and real-time monitoring should be performed before examination. Once it occurs, it should be treated immediately.						

The applicant (project leader) undertakes to:

The content filled in above is true. If approved, I will strictly abide by the "Regulation of the Research Ethics Committee of Qinhuangdao Jungong Hospital" and conduct project research according to the provided scheme.

Signature: Xiaowei Gai Date: June 15(th), 2019

Committee should be present	15 persons	Actual participating committee	15 persons	Committee agrees	15 persons

Unit review comments:

Hospital (stamped)
Date:

Attachment: brief information of the applicant (project leader)

Name	Xiaowei Gai	Gender	Female	Degree	Master	Job title	Attending physician
Telephone	15373608607	Phone number	13633335022	E-mail	gai13633335022@163.com		
Address	Department of Intensive care unit, Qinhuangdao Jungong Hospital, 15 Yufeng Road, Qinhuangdao, Hebei.	Post code	066000				
Research direction	Sepsis and Gastrointestinal Protection						
