# Supplementary material for: Risk factors for the prognosis of patients with sepsis in intensive care units
Source: PLoS One. 2022 Sep 6;17(9):e0273377. doi: 10.1371/journal.pone.0273377 (PMC9447880; doi:10.1371/journal.pone.0273377)
Supplement: Attachment — Submitted filename: PONE-D-22-04824.pdf [file pone.0273377.s004.pdf]

PLOS ONE

Risk factors for the prognosis of patients with sepsis in intensive care units

--Manuscript Draft--

**Manuscript Number:**	PONE-D-22-04824
**Article Type:**	Research Article
**Full Title:**	Risk factors for the prognosis of patients with sepsis in intensive care units
**Short Title:**	Risk factors for septic patients
**Corresponding Author:**	Qiuyan Wang<br>Qinhuangdao Jungong Hospital<br>Qinhuangdao, CHINA
**Keywords:**	Mortality; superior mesenteric artery; Ultrasound; Sepsis; case-control study
**Abstract:**	Background and purpose: To date, sepsis remains the main cause of mortality in intensive care units (ICU). This study aimed analyze the risk factors of the prognosis in sepsis patients.<br><br>Methods: In this case-control study, patients with sepsis admitted to the intensive care unit of Qinhuangdao Jungong Hospital between January and November 2020 were analyzed.<br><br>Results: A total of 72 patients with sepsis in ICU were included for analysis. The basic characteristics between the survivals and non-survivals were similar, except for acute physiology and chronic health evaluation (APACHE) II score, sepsis-related organ failure assessment (SOFA) score, lactate level, peak systolic velocity (PSV), end-diastolic velocity (EDV) and resistive index (RI). Univariate analysis revealed that the APACHE II score, SOFA score, lactate, low PSV, EDV, and RI were potential risk factors for mortality in sepsis, while multivariate analysis suggested that low PSV was an independent risk factor for mortality, and the adjusted odds ratio was 0.295 (95% CI: 0.094-0.925). The ROC analysis showed that the PSV (AUC = 0.99; sensitivity and specificity were 0.99 and 0.96, respectively) had good predictive value for mortality in sepsis, .<br><br>Conclusion: Low PSV as found to be an independent risk factor and good predictor for mortality in patients with sepsis. This study shows the promise of ultrasound in predicting mortality in patients with sepsis; however, further studies are warranted to validate these results.
**Order of Authors:**	Xiaowei Gai
	Yanan Wang
	Dan Gao
	Jia Ma
	Caijuan Zhang
	Qiuyan Wang
**Additional Information:**	
**Question**	**Response**
**Financial Disclosure**<br><br>Enter a financial disclosure statement that describes the sources of funding for the work included in this submission. Review the submission guidelines for detailed requirements. View published research articles from *PLOS ONE* for specific	This study was supported by the Medical Science Research Project of Health Commission of Hebei Province, China [20201308].The funders had no role in study design, data collection and analysis, decision to publish, or preparation of the manuscript.

Powered by Editorial Manager® and ProduXion Manager® from Aries Systems Corporation

examples.<br><br>This statement is required for submission and **will appear in the published article** if the submission is accepted. Please make sure it is accurate.<br><br>**Unfunded studies**<br>Enter: *The author(s) received no specific funding for this work.*<br><br>**Funded studies**<br>Enter a statement with the following details:<br>• Initials of the authors who received each award<br>• Grant numbers awarded to each author<br>• The full name of each funder<br>• URL of each funder website<br>• Did the sponsors or funders play any role in the study design, data collection and analysis, decision to publish, or preparation of the manuscript?<br>• **NO** - Include this sentence at the end of your statement: *The funders had no role in study design, data collection and analysis, decision to publish, or preparation of the manuscript.*<br>• **YES** - Specify the role(s) played.<br><br>* typeset	
**Competing Interests**<br><br>Use the instructions below to enter a competing interest statement for this submission. On behalf of all authors, disclose any competing interests that could be perceived to bias this work—acknowledging all financial support and any other relevant financial or non-financial competing interests.<br><br>This statement is required for submission and **will appear in the published article** if the submission is accepted. Please make sure it is accurate and that any funding sources listed in your Funding Information later in the submission form are also declared in your Financial Disclosure statement.	The authors have declared that no competing interests exist

View published research articles from *PLOS ONE* for specific examples.<br><br>**NO authors have competing interests**<br><br>Enter: *The authors have declared that no competing interests exist.*<br><br>**Authors with competing interests**<br><br>Enter competing interest details beginning with this statement:<br><br>*I have read the journal's policy and the authors of this manuscript have the following competing interests: [insert competing interests here]*<br><br>* typeset	
**Ethics Statement**<br><br>Enter an ethics statement for this submission. This statement is required if the study involved:<br><br>• Human participants<br>• Human specimens or tissue<br>• Vertebrate animals or cephalopods<br>• Vertebrate embryos or tissues<br>• Field research<br><br>Write "N/A" if the submission does not require an ethics statement.<br><br>General guidance is provided below. Consult the submission guidelines for detailed instructions. **Make sure that all information entered here is included in the Methods section of the manuscript.**	This study was approved by the Medical Ethics Committee of Qinhuangdao Jungong Hospital. All the patients voluntarily participated in the study and provided signed informed consent.

**Format for specific study types**<br><br>**Human Subject Research (involving human participants and/or tissue)**<br>• Give the name of the institutional review board or ethics committee that approved the study<br>• Include the approval number and/or a statement indicating approval of this research<br>• Indicate the form of consent obtained (written/oral) or the reason that consent was not obtained (e.g. the data were analyzed anonymously)<br><br>**Animal Research (involving vertebrate animals, embryos or tissues)**<br>• Provide the name of the Institutional Animal Care and Use Committee (IACUC) or other relevant ethics board that reviewed the study protocol, and indicate whether they approved this research or granted a formal waiver of ethical approval<br>• Include an approval number if one was obtained<br>• If the study involved *non-human primates*, add *additional details* about animal welfare and steps taken to ameliorate suffering<br>• If anesthesia, euthanasia, or any kind of animal sacrifice is part of the study, include briefly which substances and/or methods were applied<br><br>**Field Research**<br><br>Include the following details if this study involves the collection of plant, animal, or other materials from a natural setting:<br>• Field permit number<br>• Name of the institution or relevant body that granted permission	
**Data Availability**<br><br>Authors are required to make all data underlying the findings described fully available, without restriction, and from the time of publication. PLOS allows rare exceptions to address legal and ethical concerns. See the PLOS Data Policy and FAQ for detailed information.	Yes - all data are fully available without restriction

Powered by Editorial Manager® and ProduXion Manager® from Aries Systems Corporation

A Data Availability Statement describing where the data can be found is required at submission. Your answers to this question constitute the Data Availability Statement and **will be published in the article**, if accepted.<br><br>**Important:** Stating 'data available on request from the author' is not sufficient. If your data are only available upon request, select 'No' for the first question and explain your exceptional situation in the text box.<br><br>Do the authors confirm that all data underlying the findings described in their manuscript are fully available without restriction?	
**Describe where the data may be found in full sentences. If you are copying our sample text, replace any instances of XXX with the appropriate details.**<br><br>• If the data are **held or will be held in a public repository**, include URLs, accession numbers or DOIs. If this information will only be available after acceptance, indicate this by ticking the box below. For example: *All XXX files are available from the XXX database (accession number(s) XXX, XXX.*).<br>• If the data are all contained **within the manuscript and/or Supporting Information files**, enter the following: *All relevant data are within the manuscript and its Supporting Information files.*<br>• If neither of these applies but you are able to provide **details of access elsewhere**, with or without limitations, please do so. For example:<br><br>*Data cannot be shared publicly because of [XXX]. Data are available from the XXX Institutional Data Access / Ethics Committee (contact via XXX) for researchers who meet the criteria for access to confidential data.*<br><br>*The data underlying the results presented in the study are available from (include the name of the third party*	All relevant data are within the manuscript and its Supporting Information files.

and contact information or URL).<br>• This text is appropriate if the data are owned by a third party and authors do not have permission to share the data.<br><br>* typeset	
Additional data availability information:	

Powered by Editorial Manager® and ProduXion Manager® from Aries Systems Corporation

Manuscript Click here to access/download;Manuscript;Manuscript1.DOC

Risk factors for the prognosis of patients with sepsis in intensive care units

Running title: Risk factors for septic patients

Xiaowei Gai[1], Yanan Wang[2], Dan Gao[2], Jia Ma[3], Caijuan Zhang[4], Qiuyan Wang[1,*]

[1]Department of Intensive care unit, Qinhuangdao Jungong Hospital, 15 Yufeng Road, Qinhuangdao, Hebei, 066000, P. R. China

[2]Department of Operating room, Qinhuangdao Jungong Hospital, 15 Yufeng Road, Qinhuangdao, Hebei, 066000, P. R. China.

[3]Department of General surgery, Qinhuangdao Jungong Hospital, 15 Yufeng Road, Qinhuangdao, Hebei, 066000, P. R. China.

[4]Department of Anesthesiology, Tangshan Gongren Hospital, 27 Wenhua Road, Tangshan, Hebei, 064000, P. R. China Tangshan Gongren Hospital.

***Correspondence:**

Qiuyan Wang

Email: zichen0215@126.com

Tel: +8613223337716

Abstract

Background and purpose: To date, sepsis remains the main cause of mortality in intensive care units (ICU). This study aimed analyze the risk factors of the prognosis in sepsis patients.

Methods: In this case-control study, patients with sepsis admitted to the intensive care unit of Qinhuangdao Jungong Hospital between January and November 2020 were analyzed.

Results: A total of 72 patients with sepsis in ICU were included for analysis. The basic characteristics between the survivals and non-survivals were similar, except for acute physiology and chronic health evaluation (APACHE) Ⅱ score, sepsis-related organ failure assessment (SOFA) score, lactate level, peak systolic velocity (PSV), end-diastolic velocity (EDV) and resistive index (RI). Univariate analysis revealed that the APACHE Ⅱ score, SOFA score, lactate, low PSV, EDV, and RI were potential risk factors for mortality in sepsis, while multivariate analysis suggested that low PSV was an independent risk factor for mortality, and the adjusted odds ratio was 0.295 (95% CI: 0.094-0.925). The ROC analysis showed that the PSV (AUC = 0.99; sensitivity and specificity were 0.99 and 0.96, respectively) had good predictive value for mortality in sepsis, .

Conclusion: Low PSV as found to be an independent risk factor and good predictor for mortality in patients with sepsis. This study shows the promise of ultrasound in predicting mortality in patients with sepsis; however, further studies are warranted to validate these results.

Keywords: mortality; superior mesenteric artery; ultrasound; sepsis; case-control study

Introduction

Hitherto, sepsis remains the main cause of mortality in intensive care units (ICUs) [1, 2], and shock accounts for one of the highest rates of mortality, approximately 50%, in many countries [3]. Therefore, early detection and management is of chief importance. The gastrointestinal tract has long been hypothesized to play an integral role in the occurrence and development of sepsis [4]. During septic shock, the body responds by redistributing blood to vital organs, such as the heart, brain, and kidneys. However, the gastrointestinal tract is the first organ to be affected by hypoperfusion, which is considered a common indicator of the development of multiple organ failure [5, 6].

Although vasoactive drugs such as epinephrine and norepinephrine increase the perfusion pressure as well as cardiac output, they are capable of diverting blood flow away from the mesenteric circulation [5, 7], leading to the destruction of the intestinal mucosal barrier, release of inflammatory factors, intestinal epithelial hyperpermeability, bacterial translocation, and multiple organ dysfunction via the circulation [8-10] Although the underlying mechanism of total organ failure is not entirely understood, it can be avoided, since early diagnosis and management of sepsis have been associated with improved outcomes and reduced mortality [11].

The process of early detection of sepsis may be straightforward; despite this, the early stage of sepsis remains challenging for patients [11]. Routine assessments of splanchnic perfusion such as mucosal perfusion, mesenteric blood flow, mucosal oxygenation, intramucosal pH, gastric and arterial partial pressure of carbon dioxide, intestinal permeability, and lactate production can be used as preventative steps, these tools are not useful once sepsis has already

developed [12]. Therefore, there is an urgent need for an accurate, reproducible, radiation-free, rapid, and non-invasive tool to provide information about intestinal perfusion without compromising the treatment of patients with sepsis [13-15]

The use of critical care ultrasound has increased in ICUs in recent years. It has been found to be useful in identifying valuable information to improve medical decision-making, especially in patients with septic shock [16]. Ultrasound can reliably assess arterial and venous blood flow [17, 18]. The superior mesenteric artery (SMA) is the primary artery supplying the small intestine [19]. In the fasting state, approximately 25% of the cardiac output depends on the splanchnic vascular area. Postprandially, blood flow into SMA is doubled or even tripled [20]. Although effective therapy is still lacking, the therapeutic approach that is selected considerably influences mortality risk. Therefore, in the present study we hypothesized that SMA ultrasound is an effective technique to predict mortality in patients with sepsis.

Methods

Study design and patients

This case-control study involved patients with sepsis admitted to the ICU of Qinhuangdao Jungong Hospital from January 1st, 2020 to November 30th, 2020 (Date of patient recruitment, date of study and date of data collection). The authors were unable to obtain information identifying individual participants during or after data collection. The inclusion criteria were as follows: 1) patients meeting the diagnostic Sepsis 3.0 Criteria, with or without the presence of septic shock (within 1 hour of ICU admission); and 2) available SMA ultrasound within the first 24 hours of ICU admission. Patients <18 years and pregnant

women were excluded. The Research Ethics Committee of Qinhuangdao Jungong Hospital reviewed and approved of this study. All the patients voluntarily participated in the study and provided signed informed consent.

Data collection and definition

Patients data, including gender, age, APACHE Ⅱ score, SOFA score, Charlson comorbidity index (CCI), primary infection sites, disease severity (sepsis or septic shock), the use of vasopressors within 24 hour after admission, and the baseline laboratory indicators such as platelet count, C-reactive protein, lactate, and albumin levels were were acquired from medical records.

Ultrasound measurements such as the end-diastolic velocity (EDV), peak systolic velocity (PSV), and resistive index (RI = [PSV - EDV] / PSV) [14, 20] were obtained from the patient records. Two radiologists experienced in Doppler ultrasonography performed all evaluations using an ultrasonic system consisting of a 3.5-MHz sector probe (SonoSite, Bothell, WA, USA). The SMA ultrasound parameters were measured within the first 24 hours of ICU admission. The SMA measurement was obtained 1 to 2 cm proximal to the artery [21]. After acquiring a clear image of the right angle of the artery, at least five consecutive cardiac cycles were noted. Three measurements were made, and the final results are presented as the mean of the three readings [22] The prognosis of sepsis was measured by mortality of the patients.

Statistical analysis

SPSS version 26.0 (SPSS Inc., Chicago, IL, USA), GraphPad Prism version 8.0 (GraphPad,

Inc., La Jolla, California, USA), and MedCalc version 18.2.1.0 (MedCalc, Mariakerke, Belgium) software were used for statistical analysis. The measured variables are expressed as mean ± SD and the categorical values are expressed as number and percentage. The chi-square test, Fisher's exact test, Mann-Whitney *U* test, or independent Student's *t* test were used for comparisons between groups. Variables that significantly differed by the univariate analysis ($P < 0.05$) were further analyzed by the multiple logistic regression. The receiver operating characteristic (ROC) curve analysis was used, followed by calculating the area under the curve (AUC), sensitivity, and specificity. *P*-value < 0.05 was considered to be statistically significant.

Results

Among 72 patients with sepsis, there were 42 (58.3%) non-survivors and 30 (41.7%) survivors. The mean ages of the survivors and non-survivors were 71.07 ± 7.52 and 71.45 ± 6.89 years, respectively ($P = 0.822$). The mean APACHE Ⅱ and SOFA scores were higher in the non-survivors than in the survivors (APACHE II, 22.67 ± 4.28 vs. 17.43 ± 3.17 and SOFA score, 10.62 ± 1.67 vs. 7.83 ± 2.29, respectively; $P < 0.001$ for both). The lactate level showed significant difference between non-survivors and survivors (3.79 ± 1.05 mmol/L and 2.74 ± 0.92 mmol/L, respectively; $P < 0.001$; **Table 1**). The non-survivors showed lower PSV (78.92 ± 4.75 cm/s vs. 100.8 ± 7.10 cm/s), lower EDV (26.09 ± 1.51 cm/s vs. 29.55 ± 1.41 cm/s), and lower RI (0.67 ± 0.03 vs. 0.71 ± 0.02) than the survivors ($P < 0.001$ for all). However, no significant differences found in the gender, CCI score, original infection sites, occurrence of septic shock, platelet count, C-reactive protein, and albumin level between both the groups.

Compared with survivors, non-survivors experienced higher dose of norepinephrine (**Table 1**).

The statistically significant variables included APACHE Ⅱ score, SOFA score, lactate, low PSV, EDV, and RI which evidenced to be the potential risk factors for mortality by the univariate analysis (P < 0.05) were used to establish a stepwise multivariate logistic regression model. Only PSV was found to be statistically different between survivors and non-survivors; a low PSV level increased the odds ratio of mortality (OR = 0.295; 95% CI: 0.094 - 0.925; **Table 2)**.

The ROC analysis showed that the AUC of PSV (0.99) was the largest among variables of the APACHE Ⅱ score, SOFA score, lactate level, and EDV . The sensitivity, and specificity of PSV were 0.99, and 0.96, respectively (**Figure 1**).

Discussion

The present results demonstrate that low PSV was an independent risk factor for mortality, and the results of the ROC curves analyses showed that the PSV value had a good diagnostic performance in predicting mortality in sepsis, which has substantial clinical value for detection in the early stage of sepsis.

In recent years, critical ultrasound has been widely used to evaluate and to aid in the treatment of critically ill patients. Previous studies measured the blood flow velocity in SMA in several pathologies, including patent ductus arteriosus, sepsis, and necrotizing enterocolitis [23]. However, most of these assessed patients with sepsis, allowing for the application of indirect techniques to estimate visceral blood flow, but splanchnic microcirculatory blood flow has

rarely been measured [5]. We used SMA ultrasound for the evaluation of patients with sepsis and found that early monitoring of PSV and EDV is extremely valuable in predicting mortality. Although our finding can bridge the knowledge gap, it must be emphasized that our study aimed to conduct a qualitative assessment of the SMA flow, rather than an absolute quantitative comparison between the groups.

The gastrointestinal tract plays a crucial role in the pathogenesis of many diseases, and it is considered to be the motor of multiorgan failure [24]. Non-occlusive mesenteric ischemia is reported to often occur in critically ill patients or in those with significant comorbidities. It is caused by a circulatory failure as well as vasoconstriction in the mesenteric bed [19]. A previous study reported that as a result of non-occlusive mesenteric ischemia, blood flow in SMA reduced to a great extent compared to the cardiac output, and along with blood flow reduction, mesenteric vascular resistance remarkably increased [25]. Therefore, early monitoring of the mesenteric blood flow is of great importance in predicting and evaluating the outcome of severe diseases.

In this study, we observed low PSV, EDV, and RI in the non-survivor group. PSV and EDV alone showed high sensitivity and specificity in predicting the risk of mortality. Therefore, any therapeutic intervention that targets the splanchnic flow may be of great significance in critically ill patients [24]. Other approaches such as vascular catheterization, dilution techniques, and surface mucosal transducers may be more effective for monitoring blood flow; however, they are more expensive and invasive, and expose the patient to the risk of complications and radiation [14, 24]. The SMA ultrasound aids in the analysis of the superior and inferior mesenteric flow and provides several quantifiable parameters, such as pulsatility

index, RI, systolic and diastolic velocities, and blood flow volume, to assess the signal from visceral vessels [26].

Platelets play an important role in the coordinated immune response to an infection; however, during a dysregulated host response such as sepsis, platelet activation can cause inflammatory dysfunction and contribute to organ damage [27]. A previous study reported that a sharp drop in the serum albumin level due to bacteremia seriously affected the prognosis [28]. However, no significant differences were observed between the survivors and non-survivors with regard to platelet count and albumin level in our study. Lactate is a reliable prognostic biomarker for poor prognosis in patients with sepsis [12, 28]. However, the mechanism of hyperlactemia is complicated, and several reasons can cause an increase in lactate levels, including tissue hypoperfusion, underlying diseases, drugs, and birth defects. Therefore, the diagnostic value of the lactate level alone may be insufficient [28].

Despite the promising results, our study has some limitations. As only 72 elderly patients were eligible for and analyzed in our study, the study may be prone to sampling bias. This sample size may not be an accurate representation of the target population, and caution must be exercised before extrapolating the results of our study to patients with sepsis of all ages. Despite this limitation, our results are internally valid.

SOFA scores are frequently used to assess organ dysfunction and are closely related to the risk of mortality, with low scores indicating better outcomes [28]. In our study, a strong negative correlation was observed between PSV and the SOFA score; we found that the PSV value was a better predictor of mortality than the SOFA score. Based on our results, SMA ultrasound should be used at an early stage after patients with sepsis are admitted to ICU, with or without

septic shock.

Conclusion

In conclusion, low PSV and EDV values may help identify patients who are at risk of mortality. Our study provided accumulating evidence that PSV and EDV, evaluated using the SMA ultrasound, are useful to evaluate splanchnic perfusion in patients with sepsis. These findings are important for performing serial measurements in future prospective SMA ultrasounds.

Declaration of Competing Interest

The authors have no conflicts of interest to declare.

Funding

This study was supported by the Medical Science Research Project of Health Commission of Hebei Province, China [20201308]. The funders had no role in study design, data collection and analysis, decision to publish, or preparation of the manuscript.

Ethics approval

This study was approved by the Medical Ethics Committee of Qinhuangdao Jungong Hospital. All the patients voluntarily participated in the study and provided signed informed consent.

Availability of data and material

All relevant data are within the manuscript and its Supporting Information files.

References

1. Fay KT, Ford ML, Coopersmith CM. The intestinal microenvironment in sepsis. Biochimica et biophysica acta Molecular basis of disease. 2017;1863(10 Pt B):2574-83. https://doi.org/10.1016/j.bbadis.2017.03.005 PMID:28286161
2. Møller MH, Alhazzani W, Shankar-Hari M. Focus on sepsis. Intensive care medicine. 2019;45(10):1459-61. https://doi.org/10.1007/s00134-019-05680-4 PMID:31267195
3. Huan C, Lu C, Song B, Li PC, Ren HS, Chu YF, et al. The shape change index (SCI) of inferior vena cava (IVC) measuring by transabdominal ultrasound to predict the presence of septic shock in intensive care unit (ICU) patients. European review for medical and pharmacological sciences. 2019;23(6):2505-12. https://doi.org/10.26355/eurrev_201903_17398 PMID:30964177
4. Liu Z, Li N, Fang H, Chen X, Guo Y, Gong S, et al. Enteric dysbiosis is associated with sepsis in patients. FASEB journal : official publication of the Federation of American Societies for Experimental Biology. 2019;33(11):12299-310. https://doi.org/10.1096/fj.201900398RR PMID:31465241
5. Krejci V, Hiltebrand LB, Sigurdsson GH. Effects of epinephrine, norepinephrine, and phenylephrine on microcirculatory blood flow in the gastrointestinal tract in sepsis. Critical care medicine. 2006;34(5):1456-63. https://doi.org/10.1097/01.Ccm.0000215834.48023.57 PMID:16557162
6. Bartsch S, Brüning A, Reimann FM, Ludwig D. Haemodynamic effects of dopexamine on postprandial splanchnic hyperaemia. European journal of clinical investigation. 2004;34(4):268-74. https://doi.org/10.1111/j.1365-2362.2004.01323.x PMID:15086358

7. Kim HY, Kim IO, Kim WS, Kang GH. Bowel sonography in sepsis with pathological correlation: an experimental study. Pediatric radiology. 2011;41(2):237-43. https://doi.org/10.1007/s00247-010-1806-4 PMID:20734039
8. Haussner F, Chakraborty S, Halbgebauer R, Huber-Lang M. Challenge to the Intestinal Mucosa During Sepsis. Frontiers in immunology. 2019;10:891. https://doi.org/10.3389/fimmu.2019.00891 PMID:31114571
9. Li H, Zhang D, Wang Y, Zhao S. Association between acute gastrointestinal injury grading system and disease severity and prognosis in critically ill patients: A multicenter, prospective, observational study in China. Journal of critical care. 2016;36:24-8. https://doi.org/10.1016/j.jcrc.2016.05.001 PMID:27546743
10. Rowlands BJ, Soong CV, Gardiner KR. The gastrointestinal tract as a barrier in sepsis. British medical bulletin. 1999;55(1):196-211. https://doi.org/10.1258/0007142991902213 PMID:10695087
11. Mitra B, Roman C, Charters KE, O'Reilly G, Gantner D, Cameron PA. Lactate, bicarbonate and anion gap for evaluation of patients presenting with sepsis to the emergency department: A prospective cohort study. Emergency medicine Australasia : EMA. 2020;32(1):20-4. https://doi.org/10.1111/1742-6723.13324 PMID:31184442
12. Woolsey CA, Coopersmith CM. Vasoactive drugs and the gut: is there anything new? Current opinion in critical care. 2006;12(2):155-9. https://doi.org/10.1097/01.ccx.0000216584.72427.e4 PMID:16543793
13. Ukleja A. Altered GI motility in critically Ill patients: current understanding of pathophysiology, clinical impact, and diagnostic approach. Nutrition in clinical practice :

official publication of the American Society for Parenteral and Enteral Nutrition. 2010;25(1):16-25. https://doi.org/10.1177/0884533609357568 PMID:20130154

14. Maconi G, Parente F, Bollani S, Imbesi V, Ardizzone S, Russo A, et al. Factors affecting splanchnic haemodynamics in Crohn's disease: a prospective controlled study using Doppler ultrasound. Gut. 1998;43(5):645-50. https://doi.org/10.1136/gut.43.5.645 PMID:9824345

15. Nylund K, Maconi G, Hollerweger A, Ripolles T, Pallotta N, Higginson A, et al. EFSUMB Recommendations and Guidelines for Gastrointestinal Ultrasound. Ultraschall in der Medizin (Stuttgart, Germany : 1980). 2017;38(3):e1-e15. https://doi.org/10.1055/s-0042-115853 PMID:27604052

16. Chen Z, Hong Y, Dai J, Xing L. Incorporation of point-of-care ultrasound into morning round is associated with improvement in clinical outcomes in critically ill patients with sepsis. Journal of clinical anesthesia. 2018;48:62-6. https://doi.org/10.1016/j.jclinane.2018.05.010 PMID:29763777

17. Peters HP, de Leeuw D, Lapham RC, Bol E, Mosterd WL, de Vries WR. Reproducibility of ultrasound blood flow measurement of the superior mesenteric artery before and after exercise. International journal of sports medicine. 2001;22(4):245-9. https://doi.org/10.1055/s-2001-13811 PMID:11414664

18. van Bockel JH, Geelkerken RH, Wasser MN. Chronic splanchnic ischaemia. Best practice & research Clinical gastroenterology. 2001;15(1):99-119. https://doi.org/10.1053/bega.2001.0158 PMID:11355903

19. Gnanapandithan K, Feuerstadt P. Review Article: Mesenteric Ischemia. Current

gastroenterology reports. 2020;22(4):17. https://doi.org/10.1007/s11894-020-0754-x PMID:32185509

20. Byrne MF, Farrell MA, Abass S, Fitzgerald A, Varghese JC, Thornton F, et al. Assessment of Crohn's disease activity by Doppler sonography of the superior mesenteric artery, clinical evaluation and the Crohn's disease activity index: a prospective study. Clinical radiology. 2001;56(12):973-8. https://doi.org/10.1053/crad.2001.0794 PMID:11795926

21. Wicklein S, Mühlberg W, Richter B, Sieber CC. Increased splanchnic arterial vascular resistance in oldest old patients - possible relevance for postprandial hypotension. Zeitschrift fur Gerontologie und Geriatrie. 2007;40(1):37-42. https://doi.org/10.1007/s00391-007-0431-9 PMID:17318730

22. Guang Y, Ying D, Sheng Y, Yiyong F, Jun W, Shuqiang G, et al. Early Doppler Ultrasound in the Superior Mesenteric Artery and the Prediction of Necrotizing Enterocolitis in Preterm Neonates. Journal of ultrasound in medicine : official journal of the American Institute of Ultrasound in Medicine. 2019;38(12):3283-9. https://doi.org/10.1002/jum.15064 PMID:31218729

23. Maruyama K, Fujiu T, Inoue T, Koizumi A, Inoue F. Feeding interval and postprandial intestinal blood flow in premature infants. Pediatrics international : official journal of the Japan Pediatric Society. 2013;55(4):472-6. https://doi.org/10.1111/ped.12106 PMID:23566051

24. Gatt M, MacFie J, Anderson AD, Howell G, Reddy BS, Suppiah A, et al. Changes in superior mesenteric artery blood flow after oral, enteral, and parenteral feeding in

humans. Critical care medicine. 2009;37(1):171-6. https://doi.org/10.1097/CCM.0b013e318192fb44 PMID:19050615

25. Fiore G, Brienza N, Cicala P, Tunzi P, Marraudino N, Schinosa Lde L, et al. Superior mesenteric artery blood flow modifications during off-pump coronary surgery. The Annals of thoracic surgery. 2006;82(1):62-7. https://doi.org/10.1016/j.athoracsur.2006.02.012 PMID:16798189

26. Perez-Calatayud AA, Carrillo-Esper R, Anica-Malagon ED, Briones-Garduño JC, Arch-Tirado E, Wise R, et al. Point-of-care gastrointestinal and urinary tract sonography in daily evaluation of gastrointestinal dysfunction in critically ill patients (GUTS Protocol). Anaesthesiology intensive therapy. 2018;50(1):40-8. https://doi.org/10.5603/AIT.a2017.0073 PMID:29303209

27. Shannon O. The role of platelets in sepsis. Research and practice in thrombosis and haemostasis. 2021;5(1):27-37. https://doi.org/10.1002/rth2.12465 PMID:33537527

28. Gharipour A, Razavi R, Gharipour M, Mukasa D. Lactate/albumin ratio: An early prognostic marker in critically ill patients. The American journal of emergency medicine. 2020;38(10):2088-95. https://doi.org/10.1016/j.ajem.2020.06.067 PMID:33152585

Figure legends

Figure 1. The area under the receiver operating characteristic (ROC) curve (AUC). (APACHE Ⅱ: Acute Physiology and Chronic Health Evaluation Ⅱ; SOFA: Sepsis-related Organ Failure Assessment; Lac: Lactate; PSV: Peak systolic velocity; EDV: End-diastolic velocity; RI: Resistive index).

Table 1. Characteristics of septic patients (n = 72)

Characteristics	Survivors (n = 30)	Non-survivors (n = 42)	*P*-value
Age (years)	71.07±7.52	71.45±6.89	0.822
Gender			0.612
Female	11 (36.7%)	13 (31.0%)	
Male	19 (63.3%)	29 (69.0%)	
APACHE Ⅱ score	17.43 ± 3.17	22.67 ± 4.28	<0.001
SOFA score	7.83 ± 2.29	10.62 ± 1.67	<0.001
CCI score	2.33 ± 1.24	2.95 ± 1.36	0.052
Original infection sites, n (%)			0.699
Respiratory system	24 (57.1%)	20 (66.7%)	
Digestive system	10 (23.8%)	6 (20%)	
Other sites	8 (19.1%)	4 (13.3%)	
Septic shock, n (%)	11 (36.7%)	24 (57.1%)	0.087
NEmax (μg/kg/min)	0.26 ± 0.08	0.38 ± 0.08	<0.001
Platelets (×10^9/L)	156.47 ± 44.14	169.19 ± 45.91	0.243
C-reactive protein (mg/L)	91.4 ± 57.21	100.98 ± 80.13	0.577
Lactate (mmol/L)	2.74 ± 0.92	3.79 ± 1.05	<0.001
Albumin (g/L)	30.55 ± 3.34	29.09 ± 4.15	0.116
Ultrasound data			
PSV (cm/s)	100.8 ± 7.10	78.92 ± 4.75	<0.001
EDV (cm/s)	29.55 ± 1.41	26.09 ± 1.51	<0.001

RI	0.71 ± 0.02	0.67 ± 0.03	<0.001

APACHE Ⅱ: Acute Physiology and Chronic Health Evaluation Ⅱ; SOFA: Sequential Organ Failure Assessment Score; CCI: Charlson Comorbidity Index; NEmax: maxium dose of norepinephrine; PSV: Peak systolic velocity; EDV: End diastolic velocity; RI: Resistive index.

Table 2. Univariate and multivariate logistic analysis

	Univariate logistic analysis		Multivariate logistic analysis	
Characteristics	Odds Ratio (95% CI)	P-value	Odds Ratio (95% CI)	P-value
APACHE Ⅱ score	1.449（1.208,1.738）	< 0.001		
SOFA score	1.974 (1.433, 2.719)	<0.001		
Lactate (mmol/L)	2.873 (1.616, 5.108)	<0.001		
Ultrasound data				
PSV (cm/s)	0.295 (0.094, 0.925)	0.036	0.295 (0.094, 0.925)	0.036
EDV (cm/s)	0.219 (0.111, 0.432)	<0.001		
RI	<0.001 (0.001, 0.001)	<0.001		

APACHE Ⅱ: Acute Physiology and Chronic Health Evaluation Ⅱ; SOFA: Sequential Organ Failure Assessment Score; PSV: Peak systolic velocity; EDV: End diastolic velocity; RI: Resistive index; CI, confidence interval

 Click here to access/download;Figure;Figure 1.tif

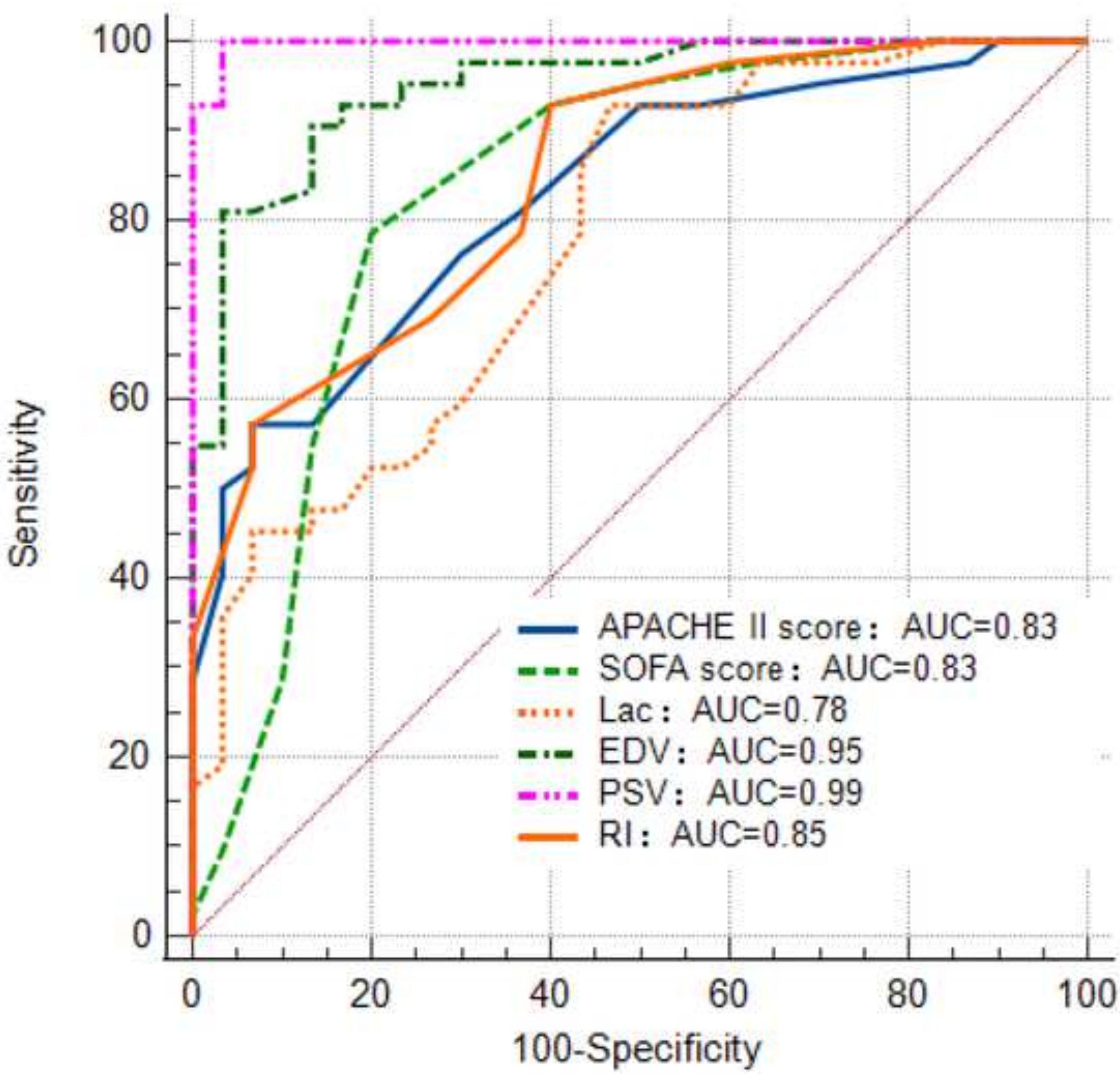

Click here to access/download
Supporting Information
Ethic approval 1.pdf

Click here to access/download
Supporting Information
STROBE-checklist-v4-combined-PlosMedicine.docx

Click here to access/download
Supporting Information
PLOSOne_Clinical_Studies_Checklist (4).docx
