# Supplementary material for: Risk factors for the prognosis of patients with sepsis in intensive care units
Source: PLoS One. 2022 Sep 6;17(9):e0273377. doi: 10.1371/journal.pone.0273377 (PMC9447880; doi:10.1371/journal.pone.0273377)
Supplement: Attachment — Submitted filename: Response to Reviewers.doc [file pone.0273377.s005.doc]

Dear Editors and Reviewers:

On behalf of all authors, I would like to express my sincere thanks for giving us the opportunity to revise our manuscript. We greatly appreciate the editors and reviewers’ positive and constructive comments and suggestions. They are all valuable and very helpful for revising and improving our paper, as well as the important guiding significance to our researches. We have studied comments carefully and have made correction which we hope meet with approval. Revised portion are marked in red in the paper. The main corrections in the paper and the responds to the reviewers’ comments are as flowing:

1. Response to comment: Is the manuscript technically sound, and do the data support the conclusions?

Response: We have revised the manuscript carefully. It describe a technically sound piece of scientific research with data that supports the conclusions.

2. Response to comment: Has the statistical analysis been performed appropriately and rigorously?

Response: We have revised the manuscript and marked in red in the paper.

3. Response to comment: Have the authors made all data underlying the findings in their manuscript fully available?

Response: We have made all data underlying the findings in the manuscript.

4. Response to comment: Is the manuscript presented in an intelligible fashion and written in standard English?

Response: We have repeatedly and carefully corrected the language in the article, striving to be clear and correct.

5. Response to comment: Review Comments to the Author.

Response to Reviewer 1: We are very sorry for our incorrect terms and words. We have revised the manuscript according to the comments and suggestions.

Response to Reviewer 2: Special thanks to the good comments. We have carefully revised the manuscript. In this case-control study, patients who met the inclusion and exclusion criteria were analyzed. Ultrasound and clinical data were analyzed and compared between non-survivors and survivors. The ROC curve analysis was also performed to determine the best indicator for predicting mortality. Two physicians experienced in Doppler ultrasonography performed all evaluations and the reliability test. The statistically significant variables that evidenced to be the potential risk factors for mortality by the univariate analysis were used to establish a stepwise multivariate logistic regression model. However, only PSV was included in the model. We have made additions and explanations in the results section.

We would like to express our great appreciation to you for your comments on our paper. We looking forward to hearing from you.

Thank you and best regards.

Sincerely,

Xiaowei Gai

E-mail: gai13633335022@163.com
